# Metabolic flux between organs measured by arteriovenous metabolite gradients

**DOI:** 10.1038/s12276-022-00803-2

**Published:** 2022-09-08

**Authors:** Hosung Bae, Katie Lam, Cholsoon Jang

**Affiliations:** Department of Biological Chemistry, Chao Family Comprehensive Cancer Center, University of California Irvine, Irvine, CA USA

**Keywords:** Metabolomics, Endocrine system and metabolic diseases

## Abstract

Mammalian organs convert dietary nutrients into circulating metabolites and share them to maintain whole-body metabolic homeostasis. While the concentrations of circulating metabolites have been frequently measured in a variety of pathophysiological conditions, the exchange flux of circulating metabolites between organs is not easily measurable due to technical difficulties. Isotope tracing is useful for measuring such fluxes for a metabolite of interest, but the shuffling of isotopic atoms between metabolites requires mathematical modeling. Arteriovenous metabolite gradient measurements can complement isotope tracing to infer organ-specific net fluxes of many metabolites simultaneously. Here, we review the historical development of arteriovenous measurements and discuss their advantages and limitations with key example studies that have revealed metabolite exchange flux between organs in diverse pathophysiological contexts.

## Metabolism: Measuring metabolite exchange between organs

Measuring concentrations of metabolites such as lipids or fatty acids in blood entering and exiting an organ reveals whether the organ uses or produces that metabolite. Organs convert food into metabolites, and specialize in producing different metabolites. These metabolites are then shared among organs; concentrations are tightly regulated, and changes can signal disease. Cholsoon Jang and coworkers at the University of California Irvine, USA have reviewed the development of techniques for measuring metabolites, highlighting key studies that illuminate metabolic roles in disease. They report that recent advances in mass spectrometry permit simultaneous measurement of hundreds of metabolites and that combining these techniques with labeled tracer molecules can reveal specific metabolite conversions within an organ. Future directions include designing less invasive methods, exploring unknown metabolites, and integration with other data, such as genomics.

## Introduction

### Exchange flux of circulating metabolites

Metabolites, the collection of small-molecule entities generated by cells, play central roles in all living organisms. Single-cell organisms such as yeast and bacteria utilize core metabolic pathways to generate metabolites for biomass and energy^1,2^. These organisms can switch on and off different metabolic pathways flexibly to produce essential metabolites for survival even in an extremely poor nutritional environment. During evolution, complex organisms lost this metabolic plasticity but instead relied on necessary metabolites from food^3^. For example, mammals cannot synthesize essential amino acids and depend solely on the diet to obtain them.

Even in the same organism, individual organs depend on each other to acquire vital metabolites. This sharing system provides tremendous benefits by distributing biosynthetic burdens and saving energy. For instance, the heart takes up circulating fatty acids derived from adipose tissues to support high energy-consuming cardiac contraction, while the brain oxidizes circulating glucose made by the liver for neuronal firing. Organs also use circulating metabolites as signaling molecules (e.g., hormones) to communicate with each other.

This mutual dependency between a dozen organs requires sophisticated regulation mechanisms to maintain circulating metabolite levels within physiological ranges. To achieve such homeostasis, organs must balance total production and consumption. Disruption in this balance can result in abnormally high or low levels of circulating metabolites, which often manifests as diseases^4^ and causes maladaptive metabolic remodeling and chronic damage to organs^5^. For example, highly elevated circulating blood glucose and lipids in diabetes cause systemic pathologies.

Over the last century, numerous studies have elucidated how organs exchange a few abundant circulating metabolites in various pathophysiological conditions. Recent revolutionary advances in mass spectrometry techniques have enabled simultaneous quantitation of hundreds of metabolites, expanding our understanding of interorgan metabolite exchange flux and its regulatory mechanisms. We will review historical and recent studies that have efficiently utilized arteriovenous (AV) metabolite measurements to reveal organ-specific metabolic fluxes. We will also discuss outstanding remaining challenges and future directions.

## Key concepts of AV measurements

### AV measurements complementing isotope tracing

**Fig. 1:**
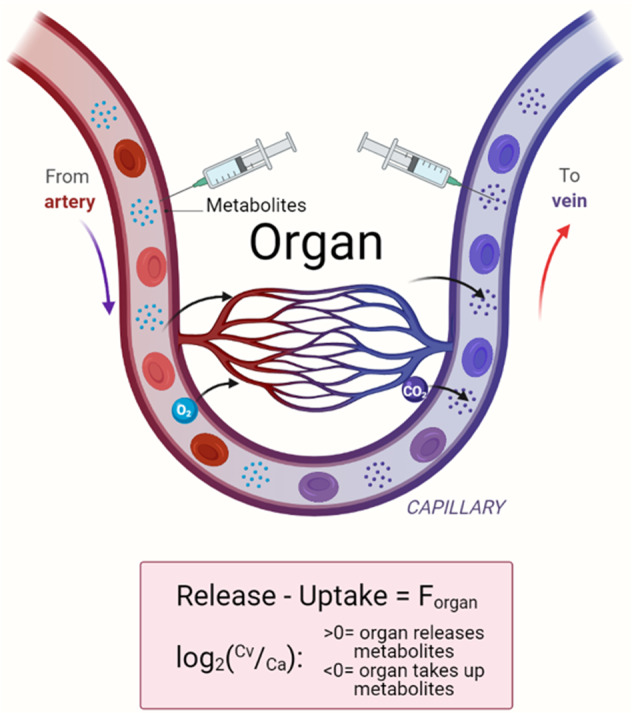
AV measurements for organ-specific metabolite exchange flux (F_organ_). The net production or consumption of metabolites by an organ can be quantified by measuring arterial (Ca) and venous (Cv) blood metabolite concentrations. If the metabolite concentration in the vein is higher than in the artery (Cv > Ca), the metabolite is net released, and in the opposite case (Cv < Ca), the metabolite is net taken up by the organ.

While multiomics organ profiling using transcriptomics, proteomics, and metabolomics provides various insights, these techniques only measure a metabolic snapshot without inferring pathway activities (or fluxes). For example, high enzyme expression or metabolite levels can be mistakenly interpreted as high flux. However, this is like thinking that many cars in a parking lot indicate rapidly moving traffic, while in fact, the opposite is true. In this regard, the most important advantage of AV metabolite gradient measurement is its ability to infer organ-specific metabolic flux (material flow per unit time) by elucidating each organ’s metabolite net uptake or release (Fig. 1).

Another way of inferring metabolic flux is to use isotope-labeled metabolite tracers (either radioactive or stable isotopes)^6–9^. Studies using isotope tracing in animals and humans have led to breakthrough discoveries in the metabolism of mammalian organs and cancer^10,11^. Isotope tracing requires adequate tracers to measure a metabolic pathway of interest. For example, ^13^C-glucose is required to study glucose metabolism, while ^13^C-lactate is required for more accurate flux calculations because glucose is rapidly converted to lactate. This interconversion between tracers and their metabolic products necessitates special mathematical modeling^12,13^. While tracers are often expensive, many groups have successfully utilized isotope tracing in large animals and humans as a discovery tool to complement AV measurements^14–16^. One key factor to consider when isotope tracers are used is the amount of tracers introduced. The amount should be sufficiently high enough to label downstream metabolites, but it should also be low enough to avoid metabolic perturbations. Nevertheless, high amounts can also be useful for studying homeostatic mechanisms^17^. Last, how the tracer is delivered into the system should be carefully considered. For example, glucose tracers can be intravenously given because they circulate at high levels, while oral delivery of fructose tracers is ideal to reflect physiological consumption and rapid catabolism by the gastrointestinal system^18–20^. In summary, the combination of AV measurements and isotope tracing is very powerful for revealing not only net production/consumption fluxes of the target organ (by AV measurements) but also the conversion of metabolites within the organ (i.e., intraorgan metabolism by comparing the labeled metabolites between the arterial and venous blood).

Due to the easy access to the small veins, AV measurements have long been performed in large animals, including rats, dogs, cats, monkeys, cows, pigs, horses, and seals. Most early studies focused on a few abundant metabolites (e.g., glucose, lactate, and amino acids) with measurements performed by biochemical enzymatic assays^21–25^. While enzymatic assays were the best available technique at that time, they can produce high data variability due to the multiple chemical reactions and washing steps. Some enzymes often exhibit promiscuity, causing overestimation of certain metabolite concentrations. To overcome these limitations, nuclear magnetic resonance (NMR) and mass spectrometry have recently become more popular for AV measurements of hundreds of metabolites simultaneously^26,27^.

### History of arteriovenous metabolite measurements

Several pioneering AV studies performed in the early 20th century have provided critical insights into the metabolic flux between mammalian organs. Carl and Gerty Cori conducted an AV study to measure glucose and lactate levels in arterial and venous blood isolated from skeletal muscle and liver. This led to their Nobel Prize discovery of the “Cori cycle”, which encompasses hepatic glucose production from muscle-derived lactate that feeds back glucose to the muscle^28^. Another discovery of the “Cahill cycle” was also achieved via AV measurements; this cycle utilizes muscle-derived alanine as fuel for hepatic glucose production^21^. To investigate other sources of hepatic glucose production, Felig et al. (1973) and Aoki et al. (1981) also used AV measurements and identified that glutamine release is increased by leucine intake, but it does not serve as an important substrate for hepatic gluconeogenesis^29,30^.

After these histologically crucial discoveries were achieved by AV measurements in humans, other researchers have adopted AV comparison to discover various metabolic fluxes in different organs in different animal species. Schwalm et al. (1971) performed AV measurements in the cow mammary gland and identified preferential uptake of circulating nonesterified fatty acids (NEFAs) over triglycerides (TGs) for milk fat synthesis during subclinical ketosis^22^. Maas et al. (1976) found in monkeys that 3-methoxy-4-hydroxyphenylethyleneglycol (a norepinephrine degradation product) is released from the head, proving its relation to the functional state of norepinephrine or serotonin neurons in the brain^23^. De Jong et al. (1977) measured the AV difference in glucose, lactate, and inosine across the pig heart to characterize the effect of local ischemia on myocardial metabolism and hemodynamics^24^. These early studies proved the advantage of AV measurements in illuminating organ-specific metabolism.

More recent AV studies have provided important insights into organ-specific metabolic flux in humans. Ivanisevic et al. (2015) performed AV comparisons at the metabolomics level in 20 human subjects and found active release of TCA cycle intermediates from the arm^26^. O’Donovan et al. (2019) measured AV differences in NEFAs, glucose, and glycerol in human adipose tissue, showing that NEFA release from adipose tissue is highly associated with the development of insulin resistance^31^. Recently, to understand human cardiac metabolism, Murashige et al. (2020) compared the heart and leg AV differences of ~300 metabolites in 110 human subjects^32^. They discovered that the failing heart (with ejection fraction <50%) takes up significantly more ketones and lactate and exhibits more active protein breakdown than the legs or the heart with preserved ejection fraction. These AV studies in humans provided enlightening evidence of clinically relevant metabolic fluxes in pathophysiological states.

### Regional blood flow measurements for flux calculations

**Fig. 2:**
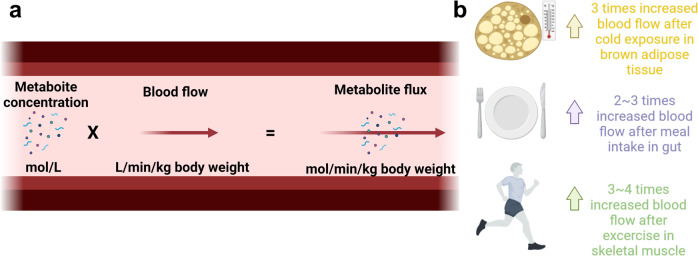
Metabolite flux calculations with blood flow measurements. **a** To quantify metabolite fluxes from AV comparisons, the blood flow, and metabolite concentrations must also be measured. **b** An increase in blood flow by cold exposure, feeding, and exercise can affect metabolic flux.

In principle, AV metabolite concentration differences reflect net uptake or release of metabolites by a measured target organ. The ratio of the venous blood to arterial blood concentration is most commonly used to indicate net uptake or net release. However, a high AV ratio does not always indicate high flux. For example, while the AV ratio is small, the flux can be large if the metabolite concentration is high or the target organ has high regional blood flow (Fig. 2a). Additionally, metabolic flux can be markedly increased by elevated blood flow even without changes in the AV ratio. For example, the gastrointestinal system after feeding, skeletal muscle after exercise, and brown adipose tissues after cold exposure are such physiological conditions^33–35^ (Fig. 2b). Disease conditions, including obesity and cardiovascular disease, can also affect the metabolite concentrations and regional blood flow rate to influence metabolic flux. Thus, accurate measurements of blood metabolite levels and regional blood flow are essential for inferring genuine metabolic flux.

Accordingly, several methods have been developed to measure regional blood flow as precisely as possible. Ultrasonic or electromagnetic instruments are the most widely used. Ultrasonic techniques include Doppler flow and transit time flow measurements. Doppler flow measures the blood velocity by detecting the transmission of the ultrasound reflecting back from the blood vessel known as the Doppler shift. However, it is limited to vessels with diameters larger than 2 mm^36^. Laustsen et al. developed the transmit time flow method that overcomes this limitation^37^. This method measures the difference in time from the transmission of an ultrasonic signal until it is received at the second transducer. Furthermore, the electromagnetic flowmeter measures an electrical signal proportional to the velocity generated by the blood as it flows through the electromagnetic field. Experiments with coronary artery bypass grafts have shown that electromagnetic flow measurements correlated with absolute flow measurements^38^. However, this technique requires the artery to be fully exposed, which potentially causes injury to the graft wall or induces vasospasm.

Indeed, arterial catheterization is quite invasive, and sampling venous output from vital organs, such as the heart, requires access to the coronary sinus located deeply inside the chest. To overcome the invasive nature of the procedure, chemical agents have been alternatively used. Injection of carbonized microspheres with radioactive isotopes allows noninvasive blood flow measurements^39,40^. Recently, nonradioactive microspheres were developed, which are ideal for chronic blood flow measurements with improved safety and lower variation compared to radioactivity decreasing over time^41^. Steady infusion of inert chemicals such as para-aminohippuric acid (PAH) is also useful to measure regional blood flow. Because PAH is not metabolized or taken up by organs, the clearance rate of PAH calculated by the AV ratio and concentrations provides the regional blood flow rate^42^. Further development and improvement in blood flow measurement techniques will greatly expand the application of AV studies for metabolic flux assessment.

## Biological insights illuminated by AV measurements

**Fig. 3:**
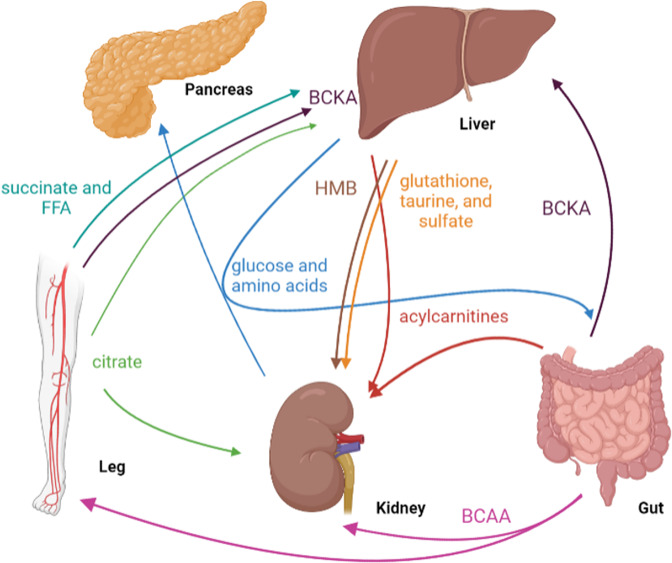
Selected examples of interorgan metabolite exchange fluxes revealed by AV comparison studies. BCAAs (branched-chain amino acids), BCKAs (branched-chain keto acids), and HMB (β-hydroxy β-methylbutyric acid).

In this section, we will discuss various biological insights discovered through AV measurements. We will describe the selected studies in two parts, physiology and pathology, in chronological order to help readers appreciate the history of AV studies (Fig. 3, Tables 1 and 2).

**Table 1:** List of AV studies that revealed metabolic fluxes in physiology.

Ref	Physiology	Condition	Species	Organ(s)	Metabolite(s)	Key findings
^48^	Fasting vs. feeding	Feeding	Rat	Liver, kidney	Methionine, cysteine, glutathione, taurine	Postprandial uptake of methionine and cysteine by the liver, possibly for glutathione synthesis. The liver released glutathione, taurine, and sulfate to the kidney for degradation.
^108^		Feeding	Pig	Liver, kidney	BCAAs, BCKAs, HMB	Postprandial exchange of branched-chain amino acids (BCAAs) and their keto acids (BCKAs) and β-hydroxy β-methylbutyric acid (HMB) by the liver and kidney
^49^		Feeding	Cow	Hindlimb	Amino acids	Postprandial uptake of amino acids by the hindlimb.
^47^		Feeding	Rat	Hindlimb	Glucose, amino acids	Postprandial uptake of glucose, glutamine, glutamate, and aspartate by the hindlimb.
^109^		Feeding	Horse	Hindlimb	Glucose, acetate, 3-hydroxybutyrate	Postprandial uptake of glucose, acetate, and 3-hydroxybutyrate by the hindlimb.
^110^		Feeding	Dog	Intestine	Glucose, fructose, galactose, lactate	Glucose, fructose, and galactose are taken up by the intestine and converted to lactate but not to glycogen
^52^		Feeding	Human	Kidney, Liver	Glucose	Increased renal and hepatic release of glucose by inducing gluconeogenesis through epinephrine.
^53^		Feeding	Human	Leg, Kidney, Liver	Glucose	There is a postprandial increase in glucose uptake by the leg and renal glucose release and suppression of hepatic glucose release.
^43^		Fasting	Human	Brain	Glucose, ketones	Upon fasting, the brain decreases glucose uptake but increases ketone uptake.
^46^		Fasting	Pig	Intestine, liver, kidney	Acylcarnitine	Upon fasting, the gut and liver release acylcarnitine, which is taken up by the kidney.
^45^		Fasting	Human	Forearm	Acylcarnitine, 3-hydroxybutyrate, acetoacetate, NEFAs	Upon fasting, the forearm takes up acylcarnitine, 3-hydroxybutyrate, and acetoacetate and releases free carnitine and NEFAs.
^27^		Fasting	Pig	11 organs	280 metabolites	Upon fasting, the liver and kidney release glucose and amino acids, which are taken up by the intestine and pancreas. The kidney is unique in that it takes up citrate.
^44^		Fasting	Human	Forearm	Leucine	Upon fasting, the forearm reduces leucine uptake
^111^		Fasting	Pig	Intestine	Lactate	Upon fasting, the intestine increases lactate release
^51^		Feed/fast cycle	Rat	Adipose tissue	Glycerol, glucose, lactate, amino acids	During fasting, the adipose tissue takes up glucose and releases glycerol and lactate. After feeding, it takes up glutamate, aspartate, glycine, and arginine and releases glutamine, serine, tyrosine, and taurine.
^50^		Feed/fast cycle	Rat	Brain	Choline	During fasting, the brain releases choline. After feeding, it takes up choline.
^57^	Different diets	High fat/high sucrose	Pig	Intestine, liver	Glucose, propionate, succinate, lactate, amino acids	High fat/high sucrose feeding increases hepatic glycogen synthesis from glucose and amino acids. The intestine takes up more propionate and succinate for gluconeogenesis.
^22^		Ketogenic diet	Cow	Mammary gland	NEFAs	A ketogenic diet increases NEFA uptake by the mammary gland.
^112^		High fat	Cow	Mammary gland	TGs, acetate, 3-hydroxybutyrate	High fat feeding increases TG and 3-hydroxybutyrate uptake by the mammary gland.
^113^		High protein	Pig	Intestine	Arginine, proline, serine, and alanine. glutamine	High protein feeding increases the intestinal uptake of amino acids.
^56^		High fat/high sucrose	Pig	Intestine	Glutamine, glutamate, choline, creatine	High fat/high sucrose feeding increases the intestinal uptake of glutamine, glutamate, choline, and creatine as alternative energy sources.
^114^	Increased energy expenditure	Exercise	Human	Leg, liver	Alanine, glutamine	Exercise increases alanine and glutamine release by the leg, and they are used by the liver to generate glucose.
^115^		Exercise	Human	Leg	Glucose, citrate, lactate	Exercise increases citrate release by the leg.
^35^		Cold exposure	Rat	Brown adipose tissue	Glucose, lactate, glycine, proline	Cold exposure increases amino acid and glucose uptake while increasing lactate, glycine, and proline release by the brown adipose tissue.
^62^		Adrenergic stimulation	Rat	Lung	Pyruvate	Under epinephrine infusion to mimic adrenergic stimulation, the lung takes up pyruvate.
^59^		Exercise	Human	Heart	Glucose, lactate	Exercise increases cardiac uptake of lactate and glucose.
^60^		Exercise	Human	Leg	Lactate	Exercise decreases net lactate release from the leg.
^61^		Exercise	Human	Brain	Lactate	Exercise increases lactate uptake in the brain.
^63^		Exercise	Human	Skeletal muscle, liver	FFAs, succinate, malate	Exercise increases free fatty acids (FFAs) 6:0 and 8:0, succinate, and malate release by the leg.
^30^		Exercise	Human	Forearm	Leucine, glutamine	Exercise or intake of a leucine meal increases forearm release of glutamine.

**Table 2:** List of AV studies that revealed metabolic fluxes in pathology.

Ref	Pathology	Condition	Species	Organ(s)	Metabolite(s)	Key findings
^31^	Metabolic syndrome	Obesity	Human	Adipose tissue	NEFAs, glycerol	In obesity, the adipose tissue takes up more glycerol and releases NEFAs.
^116^		Obesity	Human	Adipose tissue	Palmitate	In obesity, the adipose tissue takes up more palmitate upon weight loss or regain.
^117^		Obesity	Pig	Intestine, liver	Lactate, glutamate, glycine, tryptophan	In obesity, the intestine takes up more glutamate and glycine and releases tryptophan. The liver release more lactate.
^57^		Obesity	Pig	Liver, intestine	Propionate, glucose, acetate, lactate, proline, glutamate	In obesity, the liver takes up more lactate, ethanolamine, and proline and releases glutamate. The intestine releases more propionate, glucose, and acetate and takes up creatine.
^71^		Cardiomyopathy	Human	Heart	Lactate, pyruvate, glucose	In heart failure patients, upon increased arterial glucose concentration, maximal uptake of lactate, pyruvate, and glucose is observed in the heart, which is diminished as cardiac output failure is increased.
^72^		Cardiomyopathy	Human	Lung	Lactate, pyruvate, glucose	In patients with acquired heart disease, the lung takes up more lactate, pyruvate, and glucose.
^24^		Cardiomyopathy	Pig	Heart	Glucose, lactate, inosine	In myocardial ischemia, the heart takes up glucose normally but releases less lactate and inosine.
^73^		Cardiomyopathy	Human	Heart	Ketones, FFAs	In heart failure patients with reduced ejection fraction and aortic stenosis, the heart takes up more ketones and FFAs.
^74^		Cardiomyopathy	Human	Heart	Long-chain acylcarnitines	In heart failure patients with aortic stenosis and hypertrophic cardiomyopathy, the heart takes up more long-chain acylcarnitines.
^32^		Cardiomyopathy	Human	Heart	Ketones, lactate, amino acids	In heart failure patients with reduced ejection fraction, the heart takes up more ketones, lactate, glutamate, and acetate.
^66^		Diabetes	Rat	Intestine	Glutamine, glutamate, alanine	In diabetes, the intestine takes up glutamine and releases glutamate and alanine.
^68^		Diabetes	Human	Brain	Glucose, lactate, pyruvate	In insulin-dependent diabetes, the brain takes up less glucose and releases more lactate and pyruvate than the control.
^69^		Diabetes	Human	Skeletal muscle	TG	In diabetes, the skeletal muscle takes up more TGs.
^70^		Diabetes	Human	Skeletal muscle	TG	In diabetes, the skeletal muscle takes up less TGs after a polyunsaturated fatty acid meal.
^118^		Diabetes	Human	Skeletal muscle	VLDL-TG	In diabetes, the skeletal muscle takes up more VLDL-TG.
^29^		Diabetes	Human	Intestine	Leucine, glutamine	In diabetes, intestinal glutamine flux does not change compared to healthy controls.
^75^	Organ injury	Chronic Kidney Disease (CKD)	Human	Kidney	Citrulline, choline, kynurenic acid	In CKD, the kidney takes up more citrulline, choline, and kynurenic acid.
^76^		CKD	Human	Kidney	Threonine, methionine, arginine	In CKD, the kidney releases more threonine, methionine, and arginine.
^119^		CKD	Human	Kidney	Lactate, glucose, hypoxanthine	In CKD, the kidney releases more lactate, glucose, and hypoxanthine.
^77^		CKD	Human	Kidney	Purine metabolites	In CKD patients undergoing renal allograft transplantation, the kidney releases less purine metabolites.
^78^		Ischemia	Human	Kidney	Phosphocreatine, xanthine, ATP, GTP, phosphate.	In ischemia, the kidney releases more phosphocreatine, xanthine, ATP, GTP, and phosphate.
^120^		Pancreatectomy	Sheep	Placenta	Glucose	During pancreatectomy, the placenta takes up less glucose.
^121^		Cholecystectomy	Human	Liver, leg	Glucose, lactate, pyruvate, alanine, and 3-hydroxybutyrate	During a cholecystectomy, the liver takes up more glucose and the leg takes up more 3-hydroxybutyrate and releases lactate, pyruvate, and alanine.
^79^		Cirrhosis	Human	Liver	FFAs, TG, alanine, acetoacetate, 3-hydroxybutyrate	In cirrhosis, the liver takes up more gluconeogenic precursors and FFAs and releases less TG and alanine.
^80^		Sarcoidosis	Dog	Lung	Lactate, pyruvate	In sarcoidosis, the lung releases more lactate and pyruvate.
^81^		Sepsis	Human	Lung	Nitrite, S-nitrosohemoglobin	In sepsis, the lung takes up less nitrite and releases less S-nitrosohemoglobin.
^122^		Hypotension	Pig	Brain	Glucose	During hypotension, the brain takes up more glucose.
^83^		Dementia	Human	Brain	Choline	In dementia, the brain takes up more choline.
^82^		Brain Injury	Rat	Brain	Malate, phosphate, creatine phosphate	In brain injury, the brain releases more malate and phosphate and takes up more creatine phosphate.
^23^		Brain Injury	Monkey	Brain	3-methoxy-4-hydroxyphenethyleneglycol (MHPG)	In brain injury, the brain releases more MHPG.
^123^		Brain Injury	Cat	Brain	Glucose, lactate	In traumatic brain injury, the brain releases more lactate. After edema resection, the brain takes up lactate and glucose.
^124^		Brain Injury	Rat	Brain	Lactate	In traumatic brain injury, the brain releases more lactate, while sensory stimulation masked this effect.
^125^		Brain Injury	Human	Brain	Nitrite	During hypoxia-induced brain injury, the brain takes up more nitrate.
^84^		Brain Injury	Rat	Brain, liver, kidney, lower limbs, lung	Choline	During hypoxia, the lung release choline, while the brain, liver, kidney, and lower limb take up choline.
^85^		Brain Injury	Human	Brain	Lactate, glucose	In traumatic brain injury, the brain takes up more lactate and glucose.
^86^		Brain Injury	Human	Brain	Lactate	In traumatic brain injury, the net lactate release or uptake is unchanged.
^126^		Brain Injury	Human	Brain	Choline, xanthine	In traumatic brain injury, the brain releases more choline and xanthine.
^87^		Brain Tumor	Human	Brain	N-acetylornithine, glucose, putrescine, acetylcarnitine, glutamine, agmatine, and uridine 5-monophosphate.	In brain tumor patients, the tumor takes up N-acetylornithine, glucose, putrescine, and acetylcarnitine and releases glutamine, agmatine, and uridine 5-monophosphate.

### Fasting and feeding cycle

AV comparison has long been used to infer organ-specific metabolic flux during fasting. Without dietary inputs, individual organs must obtain energy from their nutrient stores (lipid or glycogen pool) or the circulation. Some organs (e.g., the brain or heart) lack such large stores and thus primarily depend on circulating metabolites. A pioneering study by Owen and Cahill et al. (1967) found a decrease in glucose uptake and a compensatory increase in ketone uptake by the brain of fasted human adults, revealing a fasting-induced shift in brain metabolism from glycolysis to ketolysis^43^. Cheng et al. (1987) and Bartlett et al. (1989) found a decreased release of leucine catabolism products and uptake of acetylcarnitine from the forearm of fasted human adults^44,45^, reflecting altered fuel sources by skeletal muscle. In fasted pigs, Schooneman et al. (2015) revealed the transfer of acylcarnitines from the gut and the liver to the kidneys, implicating active intestinal and hepatic fat oxidation^46^. Most recently, in fasted pigs, Jang et al. (2019) used AV metabolomics to map ~300 metabolites that are exchanged between 11 pig organs, revealing kidney-specific usage of circulating citrate and preferential hepatic uptake of unsaturated over saturated fatty acids^27^.

AV measurements also elucidated how different organs change their metabolic flux upon feeding. Windmueller et al. (1979) performed an AV study in the rat intestine and found a postprandial uptake of asparagine and essential amino acids and the release of nitrogen-rich metabolites (citrulline, proline, and ornithine), suggesting that intestinal nitrogen metabolism is activated by feeding^47^. Garcia et al. (1992) also used rats to show feeding-induced hepatic uptake of methionine and cysteine and the release of glutathione, taurine, and sulfate, reflecting postprandial hepatic sulfate metabolism^48^. Boisclair et al. (1993) found increased net uptake of several amino acids by the cow hindlimb, signifying enhanced protein synthesis in muscle^49^. Klein (1990) and Kowalski et al. (1997) observed feeding-induced brain choline trafficking or adipose tissue taurine and serine release, respectively^50,51^. Finally, Stumvoll et al. (1998) and Meyer et al. (2002) quantitated the altered glucose exchange between the kidney, liver, and legs after feeding^52,53^. Collectively, these AV studies highlighted distinct metabolic fluxes that occur in various organs in response to feeding.

### Dietary modifications

Diet is the major environmental factor that changes the metabolism of organs, and AV studies have been used to reveal such changes. In the cow mammary gland, Schwalm et al. (1972) detected a decreased uptake of glucose and TGs but increased uptake of NEFAs upon a switch from a ketogenic diet to a normal diet^54^. Conversely, Cant et al. (1993) found an increased uptake of TGs and ketone bodies after high-fat diet feeding for 21 days^55^. In Yucatan minipigs fed a high-fat/high-sucrose diet for two months, Poupin et al. (2019) revealed three important metabolic flux changes: (1) the liver switches from carbohydrate to lipid utilization; (2) the intestine increases its uptake of glutamine, glutamate, choline, and creatine, likely to produce more energy for nutrient digestion; and (3) the intestine also increases the protein turnover rate by increasing the uptake of several essential amino acids^56^. Under the same conditions, Tremblay-Franco et al. (2020) found increased delivery of gluconeogenic amino acids from the intestine to the liver, reflecting enhanced hepatic gluconeogenesis^57^. The liver also showed considerably increased uptake of gut microbiota-derived metabolites (e.g., propionate and succinate) and lactate as additional gluconeogenic substrates. These studies using AV measurements discovered different diet-driven alterations in organ-specific metabolic flux.

### Increased energy expenditure

Physiological conditions that require excessive energy production rewire the metabolism of organs, which can be efficiently captured by AV measurements. Wahren and Felig et al. (1971) discovered that exercised muscle substantially releases alanine and glutamine to remove excess nitrogen from amino acid oxidation^58^. In humans, during moderate-intensity exercise, Gertz et al. (1988) found increased cardiac uptake of glucose and lactate to meet the increased energy demand^59^. Intriguingly, Bergman et al. (1999) reported that after endurance exercise, circulating lactate concentrations decreased due to reduced lactate release from the leg^60^, suggesting fuel switching in muscle after prolonged exercise. In contrast to muscle, van Hall et al. (2009) showed that the brain takes up more lactate after exercise as a potential energy source^61^. Interestingly, Johnson et al. (2011) observed increased net pyruvate uptake into the lungs in response to adrenergic stimulation^62^. Such changes in an individual organ’s energy consumption can be measured by AV differences in O_2_ consumption and CO_2_ generation, as Hu et al. (2019) measured these values in each individual’s exercised leg, resting leg, and hepatosplanchnic organs of human subjects after bouts of exercise^63^.

Cold-induced thermogenesis is another physiological condition that requires high energy production. López-Soriano et al. (1988) found that rat brown adipose tissues after short cold exposure increased the uptake of amino acids and glucose and the release of lactate, glycine, and proline, indicating acute cold-induced enhanced glycolysis and amino acid oxidation^35^. In contrast, chronic cold-acclimated brown adipose tissues showed reduced lactate release but maintained high glucose consumption, suggesting full oxidation or alternative usage of glucose (e.g., fat synthesis). Additionally, the uptake of branched-chain amino acids was highly increased, while the uptake of other amino acids was reduced, reflecting selective amino acid usage by cold-adapted brown fat. Thus, AV measurements have been instrumental in identifying altered metabolic fluxes by various physiological stimuli (Table 1).

### Obesity and diabetes

AV measurements have been widely used to elucidate metabolic flux changes in organs elicited by systemic diseases (Table 2). In overweight and obese human adults, O’Donovan et al. (2019) found that caloric restriction-induced weight loss significantly increased the release of glycerol and NEFAs by adipose tissue^31^. This change was accompanied by improved insulin signaling, consistent with insulin-dependent suppression of adipose lipolysis. Polakof et al. (2018–2020) performed several AV studies in pigs fed a high-fat/high-sucrose diet and measured metabolic fluxes across the liver, gut, and adipose tissue during the development of obesity and insulin resistance^56,57,64,65^. Compared to lean pigs, obese pigs showed an overall increased transfer of gluconeogenic precursors from the intestine to the liver, highlighting the importance of the gut-liver axis during disease development.

Several AV studies have revealed how organs undergo adaptive or maladaptive metabolic remodeling in diabetes. Martin et al. (2007) compared intestinal gluconeogenesis with glutamine in streptozotocin-treated type I diabetic Sprague–Dawley rats after 72 h of fasting^66^. They detected increased glutamine uptake and gluconeogenesis by diabetic intestines. However, Brunengraber et al. (2007) did not detect increased intestinal glucose release after 48 h of fasting in hepatectomized dogs and rats^67^, suggesting that different species or durations of fasting influence the outcomes. In the brains of type 1 diabetes patients^68^, Grill et al. (1990) found a significant release of lactate and pyruvate, indicating impaired full oxidation of glucose by the brain. Hees et al. (2012) performed AV comparisons across skeletal muscle in male patients with insulin resistance^69^ and found that higher postprandial uptake of TGs by the forearm muscle and subsequently elevated intramuscular diacylglycerol levels are linked to insulin resistance. In contrast, Jans et al. (2012) identified reduced postprandial TG uptake by the muscle and increased insulin sensitivity after high dietary intake of polyunsaturated fatty acids (PUFAs)^70^, suggesting a beneficial effect of dietary PUFAs on reducing muscle lipotoxicity and insulin resistance. These studies using AV measurements revealed organ-specific metabolic flux changes by obesity and diabetes.

### Cardiovascular disease

One of the key questions in cardiovascular disease is what nutrients fuel healthy and diseased hearts, and AV measurements have contributed to answering this question. A pioneering study by Bing et al. (1953) showed increased cardiac uptake of glucose, lactate, and pyruvate in patients with heart failure^71^. Consistently, Harris et al. (1963) measured AV differences in the pulmonary and brachial arteries in patients with heart failure^72^ and found increased glucose usage by the failing heart. Voros et al. (2018) found increased uptake of ketone bodies and FFAs in both patients with heart failure/reduced ejection fraction and patients with aortic stenosis-induced left ventricular hypertrophy^73^. Pal et al. (2019) further compared substrate utilization between patients with aortic stenosis and those with hypertrophic cardiomyopathy^74^ and identified differential metabolism of long-chain acylcarnitines. As described above, Murashige et al. (2020) performed comprehensive mapping of cardiac uptake and release of numerous circulating metabolites in patients with preserved or reduced ejection fraction, which revealed that the failing heart relies on more ketones and lactate, has higher protein breakdown, and surprisingly uses almost no glucose^32^. The high protein breakdown and lack of glucose usage by the heart may reflect a fasting condition. Accurate flux calculations with direct blood flow rate measurements in these patients will greatly expand the understanding of pathophysiological cardiac metabolism.

### Organ failure

#### Kidney

AV comparison is ideal to reveal the metabolic fluxes of a select target organ. To identify metabolic fluxes that can predict future chronic kidney disease (CKD), Rhee et al. (2013) performed AV measurements of kidneys in patients who later developed CKD^75^ and discovered increased kidney uptake of 9 metabolites, including citrulline, choline, and kynurenic acid, during CKD development. Rhee et al. (2016) also performed another AV study with established CKD patients and found increased kidney release of threonine, methionine, and arginine^76^. These results suggest renal protein breakdown and the release of amino acids as potential indicators of CKD diagnosis. On the other hand, using AV measurement, Wijermars et al. (2016) studied the relationship between defective postreperfusion recovery and delayed graft function after kidney implantation^77^. In patients with delayed graft function, the researchers found continued kidney release of lactate and hypoxanthine, suggesting impaired recovery of aerobic respiration. Lindeman et al. (2020), who also studied the effects of ischemia–reperfusion injury in patients with kidney donor grafts, found xanthine release by the kidney^78^, suggesting incomplete high-energy phosphate recovery and persistent postperfusion ATP/GTP catabolism. These studies show the applications of AV comparisons in defining kidney-specific metabolism in various clinical settings.

#### Liver

Consistent with the significant roles the liver plays in regulating whole-body metabolism, impaired liver function has tremendous impacts on the metabolism of the liver itself as well as other organs. In normal subjects and cirrhosis patients with or without surgical portosystemic shunt (SPSS), Nosadini et al. (1984) examined the hepatic uptake of gluconeogenic precursors and FFAs as well as increased hepatic blood flow^79^. Compared to normal subjects, cirrhotic patients with SPSS showed lower hepatic uptake of gluconeogenic precursors and FFAs together with a lower hepatic release of TGs. Alanine release from the leg was lower in cirrhotic patients, implicating the effect of cirrhosis and surgery on systemic metabolism.

#### Lung

Respiratory dysfunction as a result of lung diseases can also alter metabolic fluxes. Strauss et al. (1970) analyzed the effects of proliferative pulmonary disease on lung metabolism in dogs^80^ and discovered that the release of lactate and pyruvate by the lung was greater than normal, potentially due to hypoxia-induced incomplete glucose oxidation. Morgan et al. (2010) examined the systemic AV differences in nitric oxide metabolites in septic patients and found that both nitrite uptake and S-nitroso-hemoglobin release by the lung are reduced by sepsis^81^.

#### Brain

Compared to other organs, there are a greater number of studies that have focused on brain metabolism using AV measurements, reflecting the relatively easy access to the jugular vein compared to other internal organ veins. Hawkins et al. (1973) studied the effect of acute ammonia toxicity on the brain in rats^82^. After injection of ammonium acetate, they observed increased uptake of glucose but not glutamine or glutamate despite the importance of these amino acids in nitrogen metabolism. Aquilonius et al. (1975) performed a brain AV study in patients with dementia^83^ and found increased choline release from the brains of the dementia groups. Interestingly, a similar observation was made by Scremin et al. (1992) in rats, which showed altered choline exchange by the brain under hypoxia^84^. They found an increase in the arterial choline concentration and the release of choline by the lungs and splanchnic territory during hypoxia, suggesting that extracerebral production of choline compensates for the brain loss of choline. While Jalloh et al. (2013) found that the human brain after traumatic brain injury (TBI)^85^ increases lactate uptake as a potential alternative energy substrate, Glenn et al. (2015) found no change in lactate trafficking under the same conditions^86^. Most recently, to identify brain tumor metabolism, Xiong et al. (2020) performed sophisticated AV metabolomics by sampling blood entering and exiting gliomas in human patients^87^. They found consumption of N-acetylornithine, glucose, putrescine, and acetylcarnitine and release of glutamine, agmatine, and uridine 5-monophosphate by gliomas. These studies using AV measurements have expanded our understanding of brain metabolism in different pathological conditions.

## Frontiers of AV metabolomics

### Minimizing anesthesia effects on metabolism

Due to the often invasive nature of the AV blood sampling procedure, most AV studies used systemic or local anesthesia. After anesthesia, a catheter can be used for repeated blood drawing from the artery or large veins (e.g., the portal vein), while needles connected to syringes can be used to collect blood from small veins. Anesthesia is known to influence organ metabolism. In addition, different anesthesia reagents can elicit distinct metabolic effects. Anesthesia can also affect regional blood flow rates^88^. To minimize the effect of anesthesia, investigators should supplement appropriate drugs/fluids/electrolytes as performed in the clinic during surgery^89^.

A less perturbative method using fluoroscopic guidance places a guide needle to visualize the optimal needle^90^. Successful guidance and blood sampling were reported for the femoral artery and vein, inferior vena cava, renal vein, jugular vein, hepatic vein, and portal vein^91–95^. While this procedure also requires anesthesia, it does not involve the opening of the abdomen or chest. Due to the lack of such complicating surgeries, this technique also allows researchers to execute longitudinal AV studies in the same animal during disease progression or long-term dietary interventions. To fully exclude the impact of anesthesia, catheters can be surgically preimplanted into the target organ vein before blood sampling in awake subjects after surgical recovery^32,57^.

### The metabolic flux between different cell types within an organ (intraorgan flux)

Since AV comparison measures ‘net’ fluxes (production minus consumption) rather than gross fluxes (total production or total consumption) across the target organ, it captures flux as a whole without information about intraorgan flux. For example, the exchange of lactate between neurons and astrocytes within the brain is not captured by the AV measurement across the whole brain, potentially underestimating total lactate flux. Likewise, AV measurements cannot distinguish metabolic flux occurring in the kidney cortex versus the medulla. Thus, in cases where the body region both consumes and produces a certain metabolite, such metabolic activity may not be detected. In some cases, certain veins drain a mixture of blood from different tissues, which makes it difficult to detect such metabolic activity of a particular organ. For example, the femoral vein draining the leg transports blood from skeletal muscle, adipose tissue, bone, and skin. This is an important point for certain metabolites that may be exchanged between tissues within the same venous bed, such as lactate exchange between adipose tissue and skeletal muscles^96^.

Encouragingly, the recent development of new technologies complements such limitations. Stable isotope tracing combined with AV measurements and mathematical modeling has been used to quantitatively measure intraorgan gross fluxes. Advancements in single-cell metabolomics may provide further granular information, although it does not infer flux^97^. While single-cell metabolomics requires cell isolation procedures, various methods to accelerate this step have been recently developed^98–100^. Finally, imaging mass spectrometry, such as matrix-assisted laser desorption ionization mass spectrometry (MALDI-MS), has played increasingly important roles in providing spatial information about metabolite abundances and distribution^101^. A combination of these techniques with isotope tracing or AV comparison will open novel avenues toward understanding metabolic fluxes at the single-cell level and spatially distinct tissue regions.

### Discovering the flux of unknown metabolites

While targeted AV metabolomics is a powerful tool to identify fluxes of annotated metabolites, there are still many unknown metabolites to be explored. In particular, the exchange flux of numerous metabolites generated by microbiota in the gut or other organs remains elusive. Additionally, our understanding of the metabolic flux of a wide range of environmentally derived metabolites, such as xenobiotics, drug metabolites, pollutants, or dietary metabolites, is still rudimentary. To elucidate such unknown metabolite uptake and release fluxes, untargeted AV metabolomics combined with various metabolite discovery/annotation tools should be employed^102,103^.

### Integrative analysis with other -omics data

Another area of future exploration is the integration of the flux data from AV metabolomics with other -omics data such as genomics, transcriptomics, proteomics, and conventional metabolomics. Such an integrative approach will be powerful to identify genes or molecules that control specific metabolic fluxes (e.g., metabolite sensors, transporters, rate-limiting enzymes). Current approaches for integrative analysis can be categorized as numerical and pathway-based. Numerical approaches include multivariate analysis such as principal component analysis or correlation-based analysis^104,105^. However, due to the complex nature of metabolic flux control (e.g., balance of substrates/products, allosteric and posttranscriptional/translational enzyme regulation), numerical approaches often fail to capture the nonlinear relationships between transcripts, proteins, and metabolic flux^106^. In addition, while pathway or network-based approaches can be used to discover specific metabolic pathways, some metabolites largely overlap with several metabolic pathways^107^ (e.g., glutamate is found in many pathways, including glutamine metabolism, neurotransmitter synthesis, glutathione synthesis, and amino acid transamination). Despite these complexities, advanced computational modeling and artificial intelligence machine learning will be useful to extract unique flux information from AV metabolomics to synergize with other -omics datasets to identify the molecular and biochemical basis of flux regulation in vivo.

## Conclusion

AV comparison studies over the last several decades have provided critical insights into distinct organ metabolism across different physiological and pathological states. From the landmark AV studies of the early 20th century that discovered the Cori cycle to the most recent advanced AV studies in multiple organs at the metabolomics levels, the power of AV measurements in illuminating biological insights that other conventional approaches can hardly detect has been exemplified. Further technological innovations and broader applications of AV measurements will expedite the identification of key metabolic fluxes that drive organ pathophysiologies, leading toward our in-depth understanding of metabolic dynamics in health and disease.
